# Reinforcement Learning Model With Dynamic State Space Tested on Target Search Tasks for Monkeys: Extension to Learning Task Events

**DOI:** 10.3389/fncom.2022.784604

**Published:** 2022-06-02

**Authors:** Kazuhiro Sakamoto, Hinata Yamada, Norihiko Kawaguchi, Yoshito Furusawa, Naohiro Saito, Hajime Mushiake

**Affiliations:** ^1^Department of Neuroscience, Faculty of Medicine, Tohoku Medical and Pharmaceutical University, Sendai, Japan; ^2^Department of Physiology, Tohoku University School of Medicine, Sendai, Japan

**Keywords:** reinforcement learning, target search task, dynamic state space, episode-dependent learning, history-in-episode architecture

## Abstract

Learning is a crucial basis for biological systems to adapt to environments. Environments include various states or episodes, and episode-dependent learning is essential in adaptation to such complex situations. Here, we developed a model for learning a two-target search task used in primate physiological experiments. In the task, the agent is required to gaze one of the four presented light spots. Two neighboring spots are served as the correct target alternately, and the correct target pair is switched after a certain number of consecutive successes. In order for the agent to obtain rewards with a high probability, it is necessary to make decisions based on the actions and results of the previous two trials. Our previous work achieved this by using a dynamic state space. However, to learn a task that includes events such as fixation to the initial central spot, the model framework should be extended. For this purpose, here we propose a “history-in-episode architecture.” Specifically, we divide states into episodes and histories, and actions are selected based on the histories within each episode. When we compared the proposed model including the dynamic state space with the conventional SARSA method in the two-target search task, the former performed close to the theoretical optimum, while the latter never achieved target-pair switch because it had to re-learn each correct target each time. The reinforcement learning model including the proposed history-in-episode architecture and dynamic state scape enables episode-dependent learning and provides a basis for highly adaptable learning systems to complex environments.

## Introduction

Learning is a fundamental process that is crucial for biological systems to adapt to the real world. Real environments have diverse states, and situation-dependent learning is indispensable to adapt successfully to such complexity. A good example of situation-dependent learning in humans is a baseball game: to win, the batter needs to bat according to the situation of the game and batting order, i.e., according to whether the previous batter got a hit and got on base. However, the batter also needs to consider of his own episode, that is, how he played against the pitcher last few times to predict what kind of ball the pitcher will throw next. An episode, which is also referred to as context in the field of neuroscience, is defined as a state (or framework) of the environment in which an agent gains experience and makes decisions or predictions (Maren et al., 2013; Yonelinas et al., 2019). Studies on episode-dependent learning provide a basis for understanding the high adaptability of living systems to real environments, and applying this to engineering.

**FIGURE 1:**
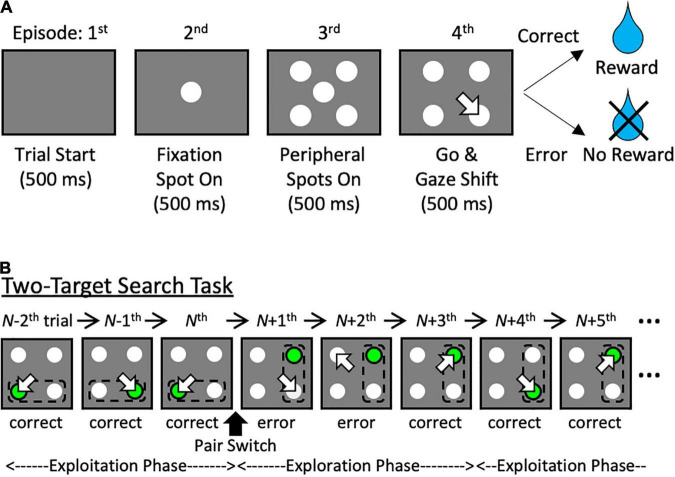
The target search task. **(A)** The event sequence of the target search task. **(B)** A similar illustration of a valid-pair switch in the two-target search task. Green circle: correct target; arrow: choice; dashed line: valid pair. Note that the subjects were not instructed to move their eyes by the green spot before gaze shift.

The two-target search task used in our non-human primate neurophysiological experiments has advantages for building models that learn behaviors based on the sequence of episodes and history of each individual episode (Kawaguchi et al., 2013, 2015). The episodes of one trial of the task are shown in Figure 1A (i.e., the sequence of task events): the central fixation spot is presented, and the animal fixates on it (2nd episode); during fixation, four light spots appear around the fixation spot (3rd episode); the disappearance of the fixation spot is used as a go signal for gaze shift to one of the four spots. If the correct light spot is fixed on, a reward is given (4th episode). To be successful in the 4th episode, an action based on the history must be selected. In the task, two adjacent light points (the target pair) among the four should be alternately selected (Figure 1B). However, after a certain number of consecutive correct responses (exploitation phase), the target pair is switched without an instruction signal, and the animal must identify a new target pair through trial and error (exploration phase). To achieve a high correct response rate in this task, action selection must be based on the history of actions and outcomes of the previous two trials.

The first model of choice for learning action while inferring what cannot be directly observed, such as a target pair, would be a reinforcement learning model using a partially observable Markov decision process (POMDP; Jaakkola et al., 1995; Thrun et al., 2005). However, applied to a two-target search task, learning models using a POMDP have *a priori* knowledge of the target pairs. Models that require such knowledge will not be able to learn unassumed tasks, as our previous studies have shown (Katakura et al., 2022). Some models do not require prior knowledge and make decisions based on history, including models involving infinite hidden Markov processes, such as the hierarchical Dirichlet process (Beal et al., 2002; Teh et al., 2006; Mochihashi and Sumita, 2007; Mochihashi et al., 2009; Pfau et al., 2010; Doshi-Velez et al., 2015). However, models using such processes do not exhibit stable performance, because they generate many useless action-value functions due to a lack of criteria regarding the appropriateness of history length required for decision-making (Katakura et al., 2022).

The reinforcement learning model with a dynamic state space that we demonstrated in our previous study does not require prior knowledge of target pairs, and adheres to criteria regarding appropriate history length, and when that length should be increased for decision-making. The model showed high performance in a two-target search task, suggesting excellent generality (Katakura et al., 2022). However, in the model described above, one trial is equal to one-time step. Thus, it cannot learn appropriate behavior in a case involving a sequence of episodes (i.e., the task event sequence shown in Figure 1A).

**FIGURE 2:**
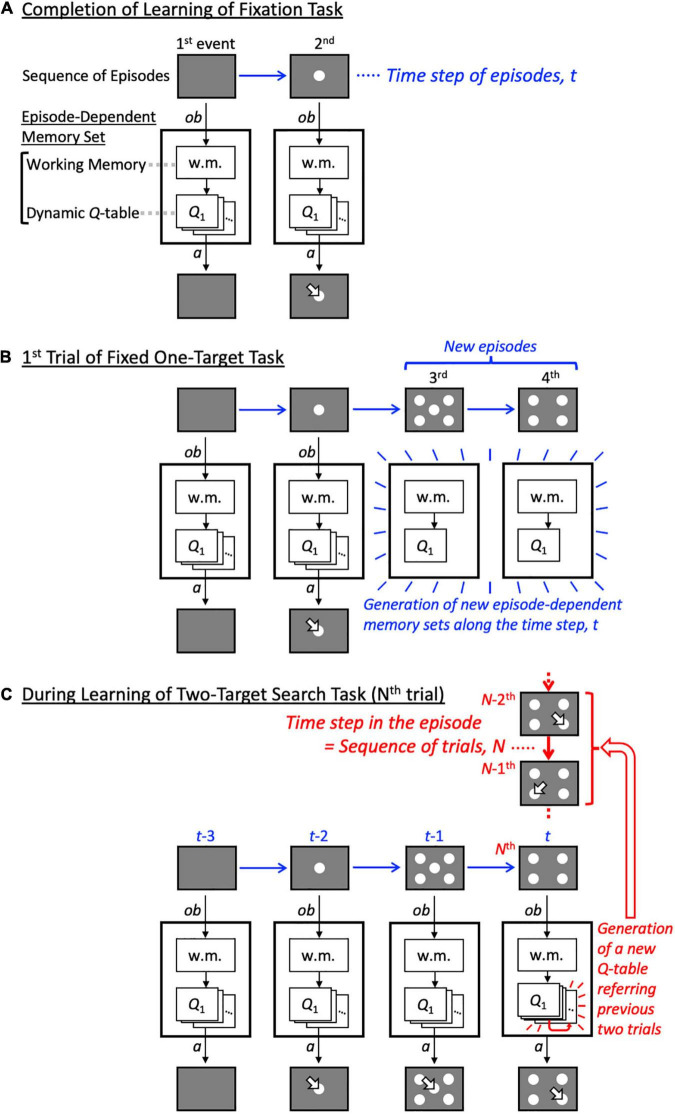
Schematic diagram of model operations as training progresses. **(A)** Schematic diagram of the model after the most elementary task, the fixation task, has been completed. Some states and corresponding *Q*-tables are generated by reflecting on the previous action and its outcome in each episode. **(B)** Schematic diagram of the model in the first trial in which the fixed one-target task was performed after the fixation task. Since the fixed one-target task includes task events 3 and 4 that the fixation task did not include, episode-dependent memory sets corresponding to task events 3 and 4 are newly generated. **(C)** Schematic diagram of the model while it is learning the two-target search task. The figure illustrates that the number of states in task event 4 is still increasing, while those in task events 1 to 3 have already stopped increasing. w.m.: working memory; arrow: choice; ob: observation; a: action.

In this study, we developed a reinforcement learning model with a dynamic state space to enable episode-dependent learning. Specifically, we added a “dynamic-state-within-episode,” or “history-in-episode,” architecture to the model. The model architecture dynamically generates a memory set when encountering a novel episode, namely, a task event (Figures 2A,B). Furthermore, the dynamic state space was used to generate a *Q*-table (action value function) for each episode (Figure 2C), according to the aforementioned criteria for appropriateness of determining state expansion: the experience saturation and decision uniqueness of action selection. These two mechanisms enable episode-dependent learning in the two-target search task. That is, the model autonomously determines that the previous state in the relevant episode is the last two trials (we refer to this as the “history” in this paper), and can find the correct new target pair in a short time without significant re-learning, resulting in high performance comparable to that exhibited by monkeys. Such learning greatly contributes to our understanding of the high adaptability of living systems to complex real environments and could lead to engineering applications.

## Materials and Methods

### Model Architecture

Our proposed model has two types of time steps/sequences (Figure 2). The first type is the sequence of episodes, *t*, on which the changes of episode, *E*_*t*_, depend. In this study, an episode is defined as a task event, specifically a display presented to an animal, rather than a sequence of events. Hence, episodes are explicit and directly observable, in the sense that the agent does not need to make any particular inferences. Temporally neighboring episodes interact when calculating reward prediction error (see below for details). The other type of step pertains to the history within an episode. Within this framework, the history at the *N^th^* trial denotes the experience with the same task event, *e*_*i*_, accrued over previous trials, and is represented as *H*_*N*_(*e*_*i*_). A given history, *h*_*j*_, is a state composed of a sequence of action–outcome pairs. Each history can include an arbitrary number of trials; however, they have a length of one trial when learning begins. Herein, we refer to this temporal structure of the model as history-in-episode architecture. The model generates a new set of memories, consisting of working memory and a dynamic *Q*-table (Figure 2A; episode-dependent memory set), when a novel task event is encountered (Figure 2B). Since our goal was to develop a learning model for a two-target search task consisting of a discrete sequence of events, the model has a simple mechanism to generate a memory set with probability 1 when a new display is exhibited. This history-in-episode architecture enables behaviors to be learned in each task period; this was not possible using the one trial-one time-step model in our previous paper (Katakura et al., 2022), in which one trial had one time unit and only the fourth task period in Figure 1A was considered.

Each episode-dependent memory set in the proposed model contained the same dynamic states as the one proposed in our previous paper (Katakura et al., 2022). The basic structure of the episode-dependent memory set was grounded in the conventional temporal difference (TD) learning (Sutton and Barto, 1998). The action value function, *Q*_*N*_(*E*_*t*_ = *e*_*i*_, *H*_*N*_ = *h*_*j*_, *A_*N*_ = a_*k*_*) for the set of a particular episode, *e*_*i*_, history, *h*_*j*_, and an action, *a*_*k*_, at the *N*th trial were updated by the following equation:


(1) $$Q_{N+1}\,\left(e_{i},h_{j},a_{k}\right)\leftarrow Q_{N}\,\left(e_{i},h_{j},a_{k}\right)+\alpha \,\delta_{t,N}\,\left(e_{i},h_{j},a_{k}\right)$$

where α is the learning rate, set to 0.1 in the range that showed desirable results revealed by the parameter search. *δ_*t,N*_* is the reward prediction error, given by


(2) $$\delta_{t,N}\,\left(e_{i},h_{j},a_{k}\right)\equiv r_{t}+\gamma \,Q_{t+1,N}\,\left(e_{i^{\prime }},h_{j^{\prime }},a_{k^{\prime }}\right)-Q_{t,N}\,\left(e_{i},h_{j},a_{k}\right)$$

where *r*_*t*_ is the reward delivered for *A*_*N*_ taken at *E*_*t*_ and *H*_*N*_ at time *t* in the *N*th trial, and the discount factor γ was set to 0.7 decided empirically. If the correct spot was selected, a reward *r* = 1 was delivered, otherwise *r* = 0 was given. *A*_*t,N*_ was selected according to the stochastic function, *P*^π^(*A_*t,N*_ = a_*k*_* | *E_*t*_ = e_*i*_*, *H_*N*_ = h_*j*_*), under the policy π. We used a softmax function for *P*^π^, defined by


(3) $$P^{\pi }\left(a_{k}|e_{i},h_{j}\right)\equiv \frac{\text{exp}\,\left(\beta \,Q\,\left(e_{i},h_{j},a_{k}\right)\right)}{\sum_{l}^{5}\text{exp}\,\left(\beta \,Q\,\left(e_{i},h_{j},a_{l}\right)\right)}$$

where the parameter β, termed the inverse-temperature, was set to 100 in the range that provided desirable results. 5 is the number of actions that the model can take. For action selection, the *Q*-table that refers to the longest history among generated *Q*-tables was used.

Our model was designed to avoid the need for stochastic decisions as much as possible. Specifically, when the model did not have a value function for a particular action that required a much larger value compared with others following extensive experience with the episode and history, it expanded the *Q*-table of the episode backward in sequence of trial (Figure 2C). We illustrate the algorithm of this expansion in Supplementary Figure 1A.

**FIGURE 3:**
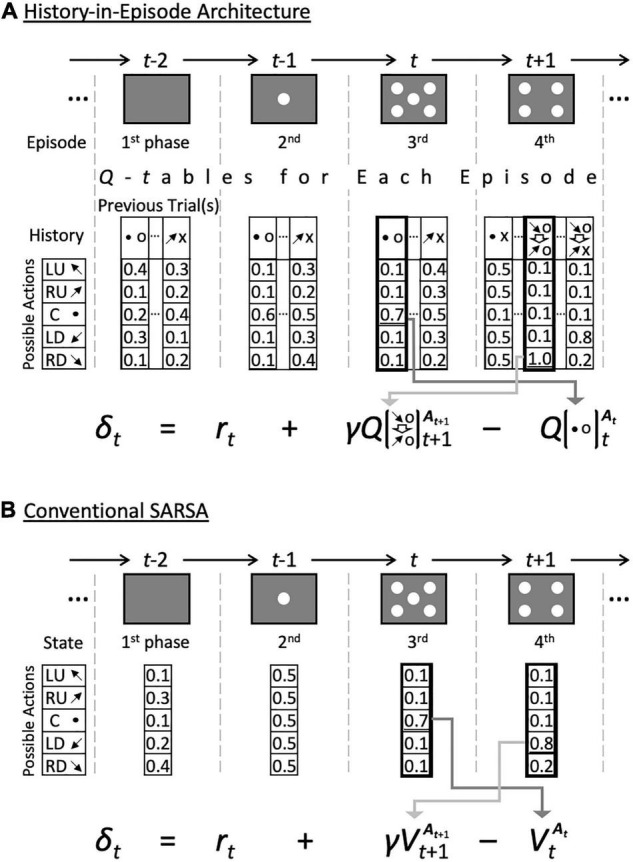
Calculation of temporary difference learning by the proposed and control models. **(A)** A history-in-episode architecture using the dynamic state model (proposed method) as an example. The action value function is selected according to the history of each episode, and the reward prediction error is calculated. Fixed 5-, 10- and 10 by 10-state models also have a history-in-episode architecture, i.e., a *Q*-table generated for each episode. However, its size does not change dynamically. **(B)** The conventional SARSA model, which is the simplest control model. Since the previous actions are irrelevant in this model, the state value function *V* is used here. This model does not have a history-in-episode architecture, as only one state-value function can be assigned to each episode.

The initial *Q-table* was set as the one of a particular combination of the five possible actions, namely gazing at the right-up (RU), left-up (LU), left-down (LD), right-down (RD) spot, or center (C), which are represented by arrows and a black dot, and the outcome (correct or error), denoted by o and x in Figure 3A and Supplementary Figure 1B. The initial *Q*-value for each action was set to 0.5. The model monitored the stochastic mean policy for each episode *e*_*i*_ and history *h*_*j*_, given by


(4) $$P_{m\,e\,a\,n,N_{u\,p\,d\,a\,t\,e,e_{i},h_{j}}}^{\pi }\,\left(\text{a}|e_{i},h_{j}\right)\equiv \frac{1}{N_{u\,p\,d\,a\,t\,e,e_{i},h_{j}}}\,\sum_{m=1}^{N_{u\,p\,d\,a\,t\,e,e_{i},h_{j}}}P_{m}^{\pi }\,\left(\text{a}|e_{i},h_{j}\right)$$

where *N_*update*, e*i*,hj_* is the number of times that the *Q*-values for the episode *e*_*i*_ and history *h*_*j*_ were updated. Then, the information gain or the Kullback-Leibler divergence (KLD) obtained by updating the stochastic policy (step 1 in Supplementary Figure 1A) is calculated:


$$U\,p\,d\,a\,t\,e\,\_\,K\,L\,D_{e_{i},h_{j}}$$

$$\left(P_{m\,e\,a\,n,N_{u\,p\,d\,a\,t\,e,e_{i},h_{j}}}^{\pi }\left(\text{a}|e_{i},h_{j}\right)||P_{m\,e\,a\,n,N_{u\,p\,d\,a\,t\,e,e_{i},h_{j}}-1}^{\pi }\left(\text{a}|e_{i},h_{j}\right)\right)$$

(5) $$\equiv \sum_{l}^{5}P_{m\,e\,a\,n,N_{u\,p\,d\,a\,t\,e,e_{i},h_{j}}}^{\pi }\,\left(a_{l}|e_{i},h_{j}\right)\,l\,o\,g\,\frac{P_{m\,e\,a\,n,N_{u\,p\,d\,a\,t\,e,e_{i},h_{j}}}^{\pi }\,\left(a_{l}|e_{i},h_{j}\right)}{P_{m\,e\,a\,n,N_{u\,p\,d\,a\,t\,e,e_{i},h_{j}}-1}^{\pi }\,\left(a_{l}|e_{i},h_{j}\right)}$$

We referred to this as the Update_KLD. *N_*update*,*ei*,hj_* – 1 indicates the number of trials since the model last encountered episode *e*_*i*_ and history *h*_*j*_ and calculated the mean *P*^π^(***a***| *e_*i*_, h_*j*_*).

Next, the model judged whether the Update_KLD of the episode *e*_*i*_ and history *h*_*j*_, fell below the criterion for experience saturation, ζ (step 2),


(6) U⁢p⁢d⁢a⁢t⁢e⁢_⁢K⁢L⁢Dei,hj≤ζ

indicating that information can no longer be gained by updating. The value of ζ was determined to be 10^–2^ in the range that showed desirable results. When the Update_KLD*_*ei,hj*_* was < ζ, the distribution of $P_{m\,e\,a\,n,N_{u\,p\,d\,a\,t\,e,e_{i},h_{j}}}^{\pi }\,\left(\text{a}|e_{i},h_{j}\right)$ was compared with $P_{i\,d\,e\,a\,l}^{\pi }\,\left(\text{a}|e_{i},h_{j}\right)$. $P_{i\,d\,e\,a\,l}^{\pi }\,\left(\text{a}|e_{i},h_{j}\right)$ is the action selection probability that only one action will be selected and was obtained as follows. First, the ideal policy, *Q*_*ideal*_(***a***| *e*_*i*_, *h*_*j*_), was obtained by setting the largest value within *Q*(***a***| *e*_*i*_, *h*_*j*_) to 1 and the other values to zero. For example, if the *Q*(***a***| *e*_*i*_, *h*_*j*_) were, {0.1, 0.4, 0.1, 0.2, 0.1}, the *Q*_*ideal*_(***a***| *e*_*i*_, *h*_*j*_), would be set to {0, 1, 0, 0, 0}.

Thereafter, the $P_{i\,d\,e\,a\,l}^{\pi }\,\left(\text{a}|e_{i},h_{j}\right)$ was calculated from *Q*_*ideal*_(***a***| *e*_*i*_, *h*_*j*_) using the softmax function in Eq. 3. For comparison, another KLD was calculated, as described below (step 3):


$$D\_KLD_{e_{i},h_{j}}\left(P_{m\,e\,a\,n,N_{u\,p\,d\,a\,t\,e,e_{i},h_{j}}}^{\pi }\left(\text{a}|e_{i},h_{j}\right)||P_{i\,d\,e\,a\,l}^{\pi }\left(\text{a}|e_{i},h_{j}\right)\right)$$

(7) $$\equiv \sum_{l}P_{m\,e\,a\,n,N_{u\,p\,d\,a\,t\,e,e_{i},h_{j}}}^{\pi }\,\left(a_{l}|e_{i},h_{j}\right)\,l\,o\,g\,\frac{P_{m\,e\,a\,n,N_{u\,p\,d\,a\,t\,e,e_{i},h_{j}}}^{\pi }\,\left(a_{l}|e_{i},h_{j}\right)}{P_{i\,d\,e\,a\,l}^{\pi }\,\left(a_{l}|e_{i},h_{j}\right)}$$

We called this the Decision-uniqueness KLD (D_KLD). When the D_KLD was below the criterion for a preference for deterministic action selection, η (step 4),


(8) $$D\,\_\,K\,L\,D_{e_{i,h}{}_{j}}<\eta$$

the agent had uniquely selected an action for the episode *e*_*i*_ and history *h*_*j*_, and the *Q*-table was not expanded any further. η was set to 2 within the range which produced fair performance revealed by the parameter search. These two criteria, ζ and η, guaranteed the appropriateness of state (history) expansion: the former is for the appropriate timing of expansion; the latter is for whether the *Q*-table should be expanded or not (Katakura et al., 2022). When the D_KLD did not meet the criterion, it was also compared to the parent D_KLD (step 5), defined as the D_KLD of the parent history from which the current history *h*_*j*_ had been expanded (e.g., Supplementary Figure 1B). In step 6, when the D_KLD is judged to be less than its corresponding parent D_KLD, as in Eq. 9,


(9) D⁢_⁢K⁢L⁢Dei,hj<P⁢a⁢r⁢e⁢n⁢t⁢D⁢_⁢K⁢L⁢Dei,hj+b⁢i⁢a⁢s

the D_KLD value is saved as the parent D_KLD, and the history is expanded as depicted in the *Q*-table of Supplementary Figure 1B (step 7). That is, the new history (child history) is the combination of the parent history and the history of one more previous trial to which the parent history refers. In the schematic example in Supplementary Figure 1B, a new history is generated from one in which the agent looked at LD and was rewarded one trial ago; this is changed to one in which it looked at LD and was rewarded one trial ago after it looked at RD and was rewarded two trials ago. The initial *Q*-value for each action is set to 0.5. On the other hand, if Eq. 9 does not hold, the current history being processed (see flowchart in Supplementary Figure 1A) is pruned (step 6’). When the current history consists of only the previous one trial, it is not erased because there is no parent history with which it could be compared. The bias is set to be −1 in all calculation.

In the current study, we compared the proposed model, including the dynamic state space, to several models with fixed state-space using the two-target search task and related simpler tasks. However, these control models also generated a new episode-dependent memory set when they encountered a novel episode or task event. The models were classified depending on the type of fixed *Q*-table in the generated episode-dependent memory set. The fixed 10-state model had a *Q*-table of size 5 by 10 in each episode, meaning that it had five action choices in each of the 10 states (histories), which were the combinations of five actions and their outcomes in the previous trial. The fixed 10 by 10-state model had states consisting of the combinations of the actions and outcomes of the two previous trials, i.e., fixed 10 by 10 states (histories). The results for this model are not shown in the current study, but this model is the optimal model when created with prior knowledge of the task structure of the two-target search task. Our previous paper (Katakura et al., 2022) showed its performance as a fixed 8 by 8-model. The fixed 5-state model obviously had five states for each episode, corresponding to the actions in the previous trial. In other words, this model did not explicitly include the result of the previous trial in the state. This model is an instrumental learning model, the results for which are omitted from the current study. The conventional SARSA model had only one value function (*V*-table, since the state was independent of the agent’s action) for each episode, and selected one action among the five choices based on the *V*-table. Therefore, this model did not include “history.” That is, while the other models contained a history-in-episode architecture (Figure 3A), the conventional SARSA model did not have that architecture (Figure 3B). It should also be noted that the conventional SARSA model is a Pavlovian learning model in which each task event serves as a CS.

### Behavioral Tasks and Simulation Framework

The target search task included the four task events “trial start,” “fixation spot on,” “peripheral spots on,” and “go & gaze shift” (Figure 1A). During the “fixation spot on” period, the agent was required to fixate on the central spot (C). In the subsequent “peripheral spots on” period, the agent was required to keep fixating on C without being distracted by the four spots presented around it: left-up (LU), right-up (RU), left-down (LD), and right-down (RD). When C disappeared at the beginning of the “go & gaze shift” period, the agent shifted its focus to one of the four surrounding spots, and if it focused on the correct target spot, it was rewarded. Note that, in Figure 1B and Supplementary Figure 2, the correct target is shown in green to help readers identify the currently correct target. In actual calculations, the agent only observe correct or error after gaze shift and cannot directly observe the true target. If the agent chose the wrong target spot, the trial was repeated under the same condition, i.e., the correct target stayed the same. The duration of each task period in the experiments with primates was 500 ms (Kawaguchi et al., 2013, 2015). In our simulations, the time step for calculation was set to one task period for simplicity.

The one-target search task (Supplementary Figure 2) was easier than the two-target task, and was used as a pretraining task for monkeys. In this task, one out of four spots served as the correct target until the target was switched to another spot after seven successive successes without the provision of additional instructions. After the target switch, the subject was required to search for the new correct target.

In the two-target search task (Figure 1B), two neighboring spots, referred to as a valid pair, were used as correct targets alternately. A valid pair was switched after seven consecutive successes without additional instructions, followed by an exploration phase for the new valid pair. Details are described elsewhere (Kawaguchi et al., 2013, 2015).

We also tested fixed one- and two-target tasks, in which the correct target or valid pair was fixed throughout the simulations, respectively, to evaluate each learning model.

### Animal Behavior

Our animal research was performed in accordance with National Institutes of Health guidelines and the guidelines of Tohoku University. All experimental protocols were approved by the Animal Care and Use Committee, Tohoku University (Permit No. ido-74). Two Japanese monkeys (*Macaca fuscata*; monkey K: 6.5 kg, monkey G: 6.1 kg) were trained to perform the two-target search task. The monkeys were kept in individual primate cages in an air-conditioned room with food available *ad libitum*. During the experiments, the monkeys sat in a primate chair with their heads restrained and faced a screen on which visual stimuli were presented. Eye position was monitored with an infrared corneal reflection system sampling at 250 Hz. Details are described elsewhere (Kawaguchi et al., 2013, 2015).

## Results

We tested the proposed dynamic state model using several behavioral tasks related to the two-target search task and compared it to other models with fixed sets of states or value functions. This comparison revealed fundamental differences between the compared models.

**FIGURE 4:**
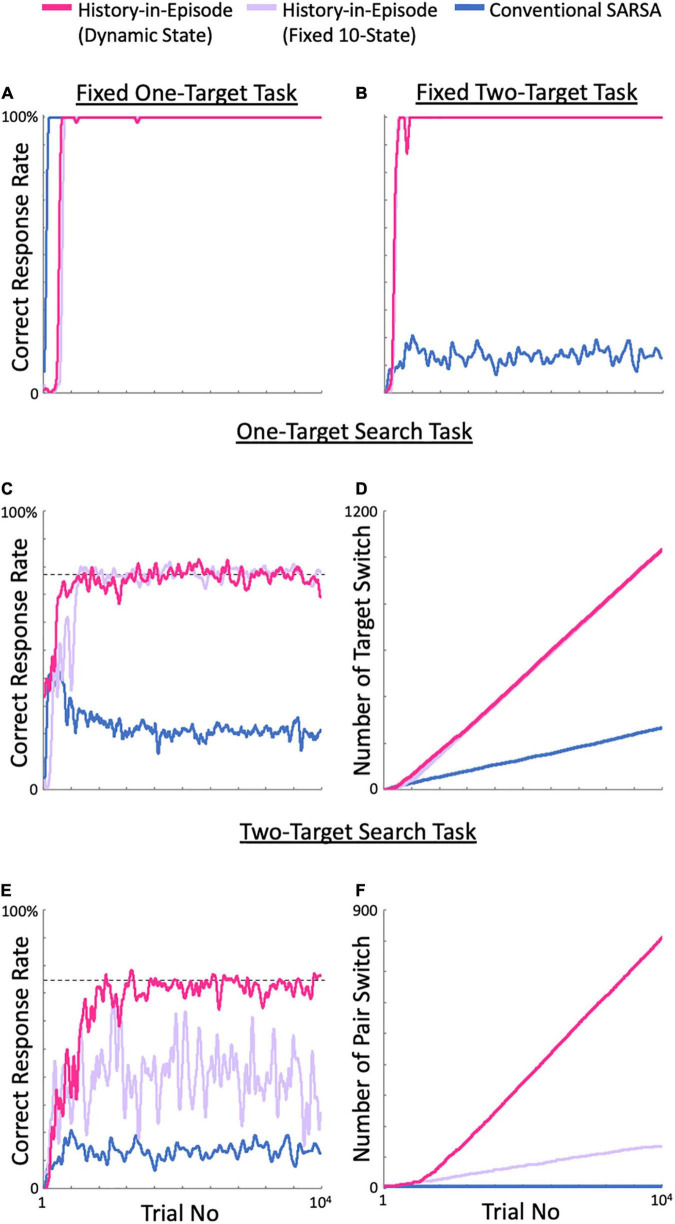
Comparison of the performance on each task between the proposed and control models. **(A)** Evolution of the correct response rate in the fixed one-target task. **(B)** The fixed two-target task. **(C)** One-target search task. Dashed line: ideal performance. **(D)** Evolution of the number of target switches. **(E)** Two-target search task. Dashed line: ideal performance. **(F)** Number of valid-pair switches. All calculations started at the initial state.

First, we tested all models using a fixed one-target search task with only one correct target spot during the entire simulation. All models exhibited almost perfect performance (Figure 4A; data not shown for the fixed 10 by 10- and 5-state models. The same applies to the following results). However, it is noteworthy that the simplest model, i.e., the conventional SARSA model, learned the fastest.

Figure 4B shows the results of the fixed two-target task. In this task, the correct valid target pair was not changed during the entire simulation, but two targets in the pair were the correct target alternately. This setup created additional difficulty since the correct strategy in the previous trial is not valid, and the models had to switch their behavior alternatively depending on the state, i.e., the history. Under these conditions, we expected the conventional SARSA model to exhibit poor performance because it was not able to make decisions based on the previous actions. As expected, all models except the conventional SARSA model showed almost perfect performance.

The one-target search task revealed additional differences between the tested models (Figures 4C,D). This task required the agent to adapt to a switched correct target after every seven consecutive successes. This requirement forced the conventional SARSA model, as well as the fixed 5-state model (data not shown), to re-learn the correct target after each switch. As a result, they exhibited much lower correct response rates (Figure 4C) and numbers of target switch (Figure 4D) than the dynamic state, fixed 10- and 10 by 10-state models. These superior models, in contrast, learned how to explore in the exploration phase after a target switch, because the state, i.e., history, explicitly included the previous outcome as well as the action, which led to almost ideal performance (dashed line in Figure 4C), although some delay in the increase in correct response rate was observed for the fixed 10-state model.

Finally, we tested all models on the two-target search task (Figures 4E,F). As expected, our dynamic state model reproduced the results of our previous paper (Katakura et al., 2022), and showing nearly ideal performance (dashed line in Figure 4E) and a high number of pair switches (Figure 4F); the same performance was obtained for the fixed 10 by 10-state model (data not shown), which was created as an ideal model with prior knowledge of the task structure. As for the fixed 10-state model, although it performed well for the one-target search task, its performance for the two-target search task was much worse than the ideal performance. This poor performance was expected because the model included only one previous trial in its history, while the ideal performance required inclusion of the two previous trials in its history. The fixed 5-state model showed similar performance to the fixed 10-state model. The conventional SARSA model exhibited a lower correct response rate than in the one-target search task and achieved no pair switch. Re-learning to focus on each of the spots of the valid pair never allowed the conventional SARSA model to achieve a pair switch.

**FIGURE 5:**
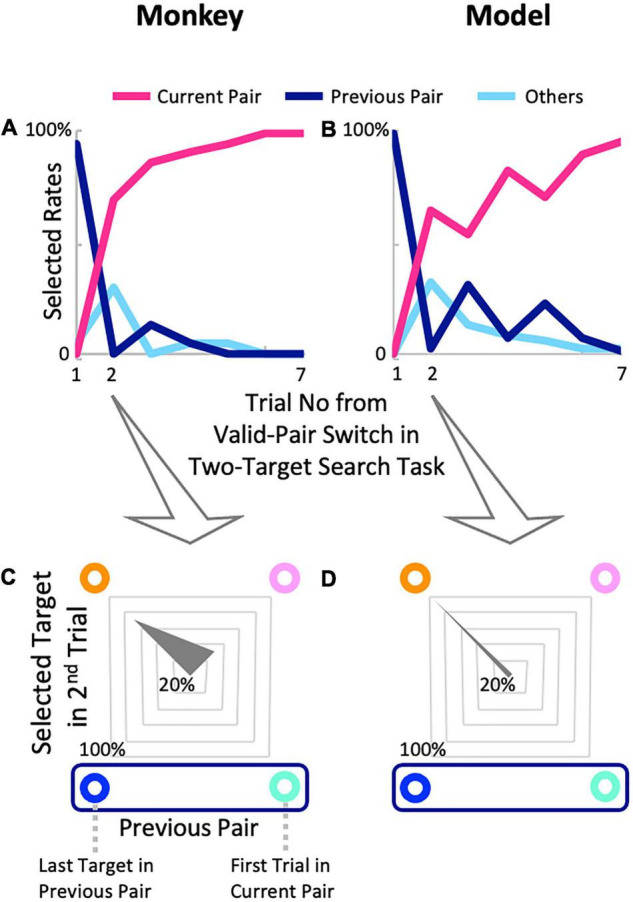
Comparisons of model performance and the exploratory behavior of a monkey after valid-pair switches in the two-target search task. **(A)** Monkey G’s exploratory behavior while recording the activity of a neuron (20509U1a2) after completing the training; there were 23 pair switches. **(B)** Exploration behavior of the model, with 774 pair switches. The gaze direction distributions of the monkey **(C)** and model **(D)** in the second trial, after switching the valid pairs.

The proposed model performed as well as a monkey in the two-target search task (Figure 5). The monkey quickly located new valid pairs after valid-pair switches (Figure 5A). Since valid pairs were switched without any explicit instruction, he inevitably gazed at the target of the previously valid pair in the first trial of the exploration phase (dark blue line in Figure 5A), whereas he was highly likely to gaze at the new pair target after the first trial (red line in Figure 5A). The rapid switching to the new pair displayed by the monkey was also seen in the proposed model (Figure 5B). Furthermore, to examine the exploratory behaviors of the monkey and model in detail, gaze directions in the second trials of the exploration phase were analyzed, and we found that both the monkey (Figure 5C) and model (Figure 5D) were highly likely to gaze at the target diagonal to the one in the first trial (orange circles in Figures 5C,D). These results indicate that the early detection of new pairs is achieved by sophisticated, non-random exploratory behavior.

**FIGURE 6:**
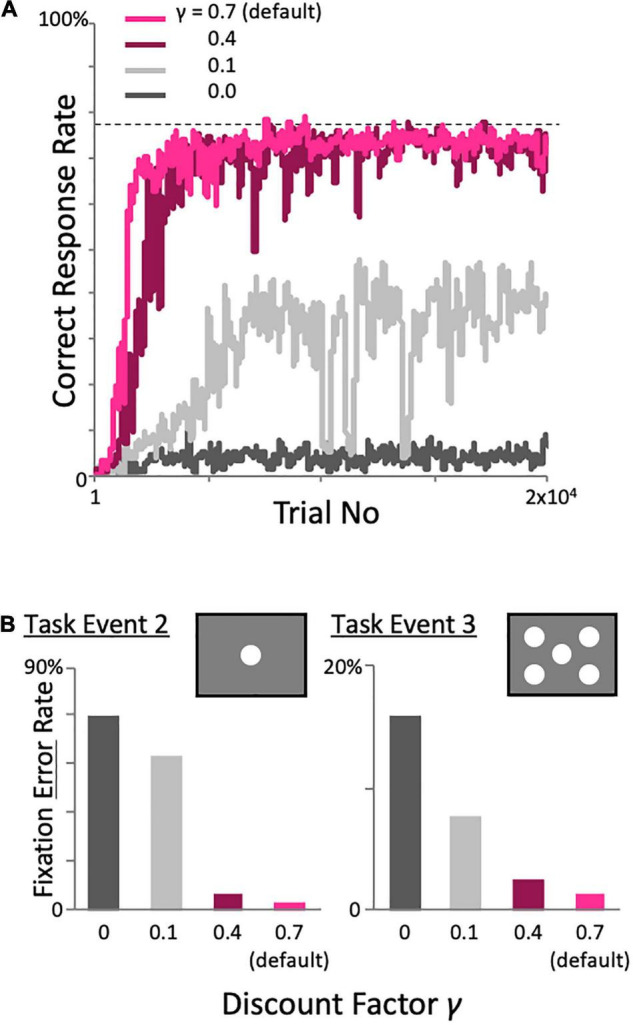
Effects of varying the discount factor on performance in the two-target search task. **(A)** Changes in the percentage of correct trials with learning. The incorrect response rates during task events 2 **(B)** The incorrect response rates during task events 2 and 3 when varying the discount factor.

Previously, we showed that good performance can be achieved over a wide range of meta-parameter, i.e., the learning rate, inverse temperature, threshold of experience saturation, and threshold of decision uniqueness, through parameter search (Katakura et al., 2022). Here, we examine model performance while varying the discount factor Eq. 2, which was not included in our previous one trial-one time-step model (Figure 6). When the high default value of 0.7 was reduced to 0.4, the model achieved a high correct response rate, although learning was relatively slow. However, when the default value was reduced further, the performance deteriorated rapidly (Figure 6A). This deterioration was not due only to the selection of the correct target in task event 4, but also to the inability to maintain fixation in the preceding task events. When the discount factor was reduced, the fixation error rate in each task event, i.e., the percentage of trials in the task event of interest that had fixation errors relative to the total number of trials on which task performance was maintained up to that task event, increased. In addition, the error rate in task event 3 was lower than that in task event 2, which is remote from task event 4 (in which the reward is actually delivered; Figure 6B). This implies that a high discount factor is required to learn a task involving a long sequence of events with a reward given only at the end of a trial.

Executing the two-target search task with a high correct response rate requires making decisions based on the actions of the previous two trials and their outcomes. However, this is only true for the action selection during task event 4. Other task events require the agent to only fixate to the central spot. The dynamic state model learns to execute the task while increasing the states consisting of actions and their outcomes. However, when learning to focus on only one spot regardless of the previous actions, learning using a single state, i.e., Pavlovian learning, might not only be sufficient, but could even speed up learning.

**FIGURE 7:**
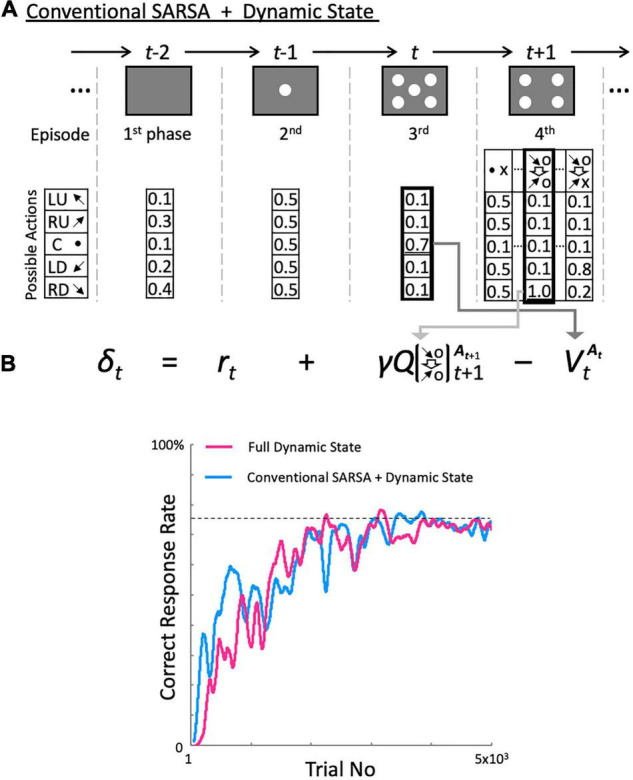
Configuration and performance of the hybrid model. **(A)** Calculation of reward prediction error in the hybrid model. Dynamic state space is given only to task event 4. **(B)** Performance comparison between the hybrid and full dynamic state models (Figure 3A) in the two-target search task. All calculations started at the initial state.

To test this idea, we implemented a hybrid model in which we used a single *Q*-table for task events 1 to 3 and a dynamic *Q*-table for only task event 4 (Figure 7A). Figure 7B compares performances between the dynamic state and hybrid models on the two-target search task. Almost ideal correct response rates were obtained (dashed line in Figure 7B); however, the performance of the hybrid model increased earlier than that of the dynamic state model. These results support our idea that, by minimizing the number of states when learning how to fixate on the center spot, the hybrid model speeds up its learning during the first three task events.

To further confirm this, we developed a parallel model in which Pavlovian, fixed 5-state, and dynamic state models were calculated in parallel for each episode and an action was selected based on the *Q*-table exhibiting the highest decision uniqueness among the three models. After executing 10,000 trials, we examined the model used in each task event and found that the dynamic model was used in task event 4, while the Pavlovian model was used in the other three task events. This result indicated that the most appropriate learning model changes depending on the task requirements.

## Discussion

In this study, we proposed a history-in-episode architecture to extend a reinforcement learning model, enabling episode-dependent learning. In addition, we built a model that also included the dynamic state space proposed in our previous paper (Katakura et al., 2022), and tested its performance in a two-target search task. By having episode and history, the model was able to learn the appropriate action for each event in one trial based on the history of recent trials. The proposed model, which includes the dynamic state space and the history-in-episode architecture, is expected to be further developed and applied as a pioneering learning model with high adaptability to complex real environments, since it learns appropriate behaviors under various circumstances.

As shown in our previous paper (Katakura et al., 2022), the dynamic state model had a sufficient range of well-behaved meta-parameters for its intrinsic parameters, such as experience saturation and decision uniqueness, as well as conventional parameters such as learning rate and inverse temperature of the softmax function for action selection. This robustness was also true for the model with the history-in-episode architecture presented in the current study. Unlike the one trial-one time-step model in our previous paper, the model including the history-in-episode architecture uses TD learning to learn the task events. In TD learning, the discount factor is used as a coefficient that is multiplied by the reward prediction at the next time step in calculating the reward prediction error in Eq. 2. The model exhibited desirable performance in a sufficiently wide range of discount factors as shown in Figure 6. When the discount rate was too low, TD learning was unsuccessful and the model did not learn to take any action, specifically not during the earlier task events. The desirability of a high discount rate is also consistent with Go and Shogi models (Silver et al., 2016, 2017), which learn behavior for long and complex orders of steps.

In recent years, machine learning and artificial intelligence (AI), as exemplified by learning models for Go and Shogi, have outperformed humans in some tasks (Silver et al., 2016, 2017). However, it is questionable whether these models can be implemented in field robots working in real environments. Although the models can outperform humans in a single task, they lack some basic structures that are crucial for flexible learning in a real environment with complex situations and multiple goals. As shown in Figure 4, when multiple targets must be achieved (fixed two-target task), or when targets are frequently switched (one-target search task), the conventional SARSA or Pavlovian model or exhibited poor performance. In contrast, the fixed 10-state model with history-in-episode architecture achieved high performance for these two tasks, although state space was fixed. Even the fixed 5-state, i.e., the conventional instrumental learning model, which did not explicitly include the outcome of the previous trial, showed high performance in the fixed two-target task by choosing an action depending on the action in the fourth task period of the previous trial. Therefore, the history-in-episode architecture proposed in the current study provided a framework for achieving multiple goals, which has recently been a research issue (Bai et al., 2019; Colas et al., 2019; Zhao et al., 2019; Pitis et al., 2020; Shantia et al., 2021).

However, that does not mean that Pavlovian learning is always inferior. Our proposed model dynamically generated states and corresponding *Q*-tables based on combinations of actions and their outcomes. However, in task events 1 to 3 of the two-target search task, it is sufficient to simply learn to fixate, and having multiple states in each task event seems redundant. The hybrid model, which learns in a Pavlovian fashion in all task periods except the fourth using only a single *V*-table, learned the task faster than the full dynamic model (Figure 7B). A similar observation is shown in Figure 4A: for the fixed one-target task, the conventional SARSA model with Pavlovian learning during all task periods learned the task faster than the other models. These computational examples show that when states are redundant, the frequency with which each state is encountered decreases, resulting in slower learning. These arguments are related to the debate about whether Pavlovian or instrumental learning is better (Rescorla and Solomon, 1967), and how they can be used differently (Cartoni et al., 2016). Dorfman and Gershman (2019) developed a model in which either Pavlovian or instrumental conditioning predominated, depending on the degree to which an action can control the reward. We also generated a parallel model that included Pavlovian, instrumental, and dynamic state models, computed them in parallel, and let it select an action via the model exhibiting the highest decision uniqueness. We found that in the two-target search task, the Pavlovian model was used in task events 1 to 3, which are independent of the previous action. These observations suggest that learning models that are as simple as possible, i.e., having only the necessary states, are preferable. Choosing a resource-saving learning method according to the task requirements can avoid the curse of dimensionality problem in reinforcement learning (Sutton and Barto, 1998) and increase the learning speed.

If an action and its outcome are not uniquely predicted, it is desirable to increase the number of states so that the action and outcome can be uniquely expected by incorporating new clues. When presented with an ambiguous CS, i.e., when a US follows a CS in an episode or experimental condition but not in another condition, rats can uniquely predict the US by considering the information available under each condition, i.e., some clues in the environment or the configuration between them (Fanselow, 1990). The first brain region that contributes to such episode-dependent learning is the hippocampus. For example, hippocampal lesions in rodents produce deficits in freezing behavior during exposure to a shock-paired condition (Selden et al., 1991; Kim and Fanselow, 1992; Phillips and LeDoux, 1992). The structure and function of the hippocampus should be taken into account when developing our proposed model into one more in line with the structure of the real brain.

Some readers may find similarities between assigning a different *Q-*table to each episode in our model and learning sub-tasks in hierarchical reinforcement learning (HRL) models (Barto and Mahadevan, 2003; Hengst, 2010; Al-Emran, 2015; Pateria et al., 2021). However, since the two-target search task has temporally discrete task events, we need only generate a new episode-dependent memory set when a new task event is presented, and avoid the difficult problem of generating sub-tasks by deciding how to divide a continuous scene, which is one of the main issues for HRL. Moreover, our model is not hierarchical in the same sense of HRL. That is, our model does not include a supervisor that overlooks the units learning the sub-tasks, and gives them sub-goals. For these reasons, our model is not meant to be considered alongside or compared with HRL models. Rather, the proposed model includes two types of time steps i.e., the time step across different task events and the dynamic history in the episode of interest, and has a structure that generates memory sets or *Q*-tables as required in each time direction, especially in the case of history, where the state is generated dynamically to refer to multiple steps in the past. We consider these to be two novel points of the proposed model, and to be indispensable for learning the two-target search task. In our previous physiological studies, we observed neuronal activities in the lateral prefrontal cortex of monkeys that reflected sub-task generation (Saito et al., 2005; Mushiake et al., 2006; Sakamoto et al., 2008, 2013, 2020a). In the future, HRL will have to be considered when modeling those neural activities.

Other route to improvement of our proposed model is the involvement of finer time and space increments. To train a monkey to perform the two-target search task, it is necessary to start with the fixation task, in which the monkey is required to fixate on a single point in a continuous wide field of view and then complete simple tasks, such as the one-target search task used in the current study. In addition, it is necessary to gradually increase the length of each task period and gradually decrease the number of trials required to switch between targets or valid pairs in the pretraining trials. In contrast, in our computer simulations, the dynamic state model was able to learn the two-target search task without any pretraining. This is because we made the conditions of the simulations as simple as possible: one task period corresponded to one time step in the calculation and there were only five discrete choices of actions. In future, when the model becomes applicable to finer increments of time and space, the training of the model will require steps compatible to the training of monkeys.

However, it remains difficult to determine appropriate training steps. We have trained monkeys to perform many advanced behavioral tasks (Mushiake et al., 2001; Sakamoto et al., 2008, 2013, 2015, 2020a,b) and obtained empirical knowledge regarding appropriate training steps. This knowledge is crucial for effective training. A major focus for future work will be to determine appropriate training steps for the model. We will aim to develop a “coach” that outputs task parameters, such as the length of the task period or complexity of the task, depending on the task conditions and learner’s behavior, etc. The coach, which also needs to be equipped with a model involving a dynamic state space and a history-in-episode architecture, and the learner then start co-learning. In developing such a coach, the main challenge will be formulating the task complexity and generating a new training step depending on the progress of the learner. However, if we can generate such a coach model, it will likely be the prototype of a new type of AI that co-develops with humans and draws out our potential abilities rather than “confronting” us. Such a system could be referred to as hyper-adaptable, and we hope to use such systems to create a new discipline called neuro-coaching (Sakamoto, 2019).

## Data Availability Statement

The raw data supporting the conclusions of this article will be made available by the authors, without undue reservation.

## Ethics Statement

The animal study was reviewed and approved by the Animal Care and Use Committee, Tohoku University (Permit No. ido-74).

## Author Contributions

KS designed the research, analyzed the data, wrote the first draft of the manuscript, edited the manuscript, and wrote the manuscript. KS and HY performed the research. NS and HM designed the two-target search task. YF obtained the behavioral data. KS and NK analyzed the behavioral data. All authors contributed to the article and approved the submitted version.

## Conflict of Interest

The authors declare that the research was conducted in the absence of any commercial or financial relationships that could be construed as a potential conflict of interest.

## Publisher’s Note

All claims expressed in this article are solely those of the authors and do not necessarily represent those of their affiliated organizations, or those of the publisher, the editors and the reviewers. Any product that may be evaluated in this article, or claim that may be made by its manufacturer, is not guaranteed or endorsed by the publisher.

## Supplementary Material

Supplementary Figure 1 Expansion and contraction of the history. **(A)** Flowchart of the expansion and contraction process. **(B)** An example of expansion of a history derived from the parent history in *Q*-table. The direction of the arrow represents the target that the agent looked at, and o and x represent the correct answer and error, respectively. The example in the figure shows that a new history is generated from the history that the agent looked at LD and was rewarded one trial ago, to the history that it looked at LD and was rewarded one trial ago after it looked at RD and was rewarded two trials ago. The numbers in the *Q*-table represent *Q*-values. The initial *Q*-value for each action is set to 0.5.

Supplementary Figure 2 A schematic example of a target switch in the one-target search task. The format is the same as the task shown in Figure 1B.
