# Supplementary figures and images for: Reinforcement Learning Model With Dynamic State Space Tested on Target Search Tasks for Monkeys: Extension to Learning Task Events
Source: Front Comput Neurosci. 2022 Jun 2;16:784604. doi: 10.3389/fncom.2022.784604 (PMC9201426; doi:10.3389/fncom.2022.784604)

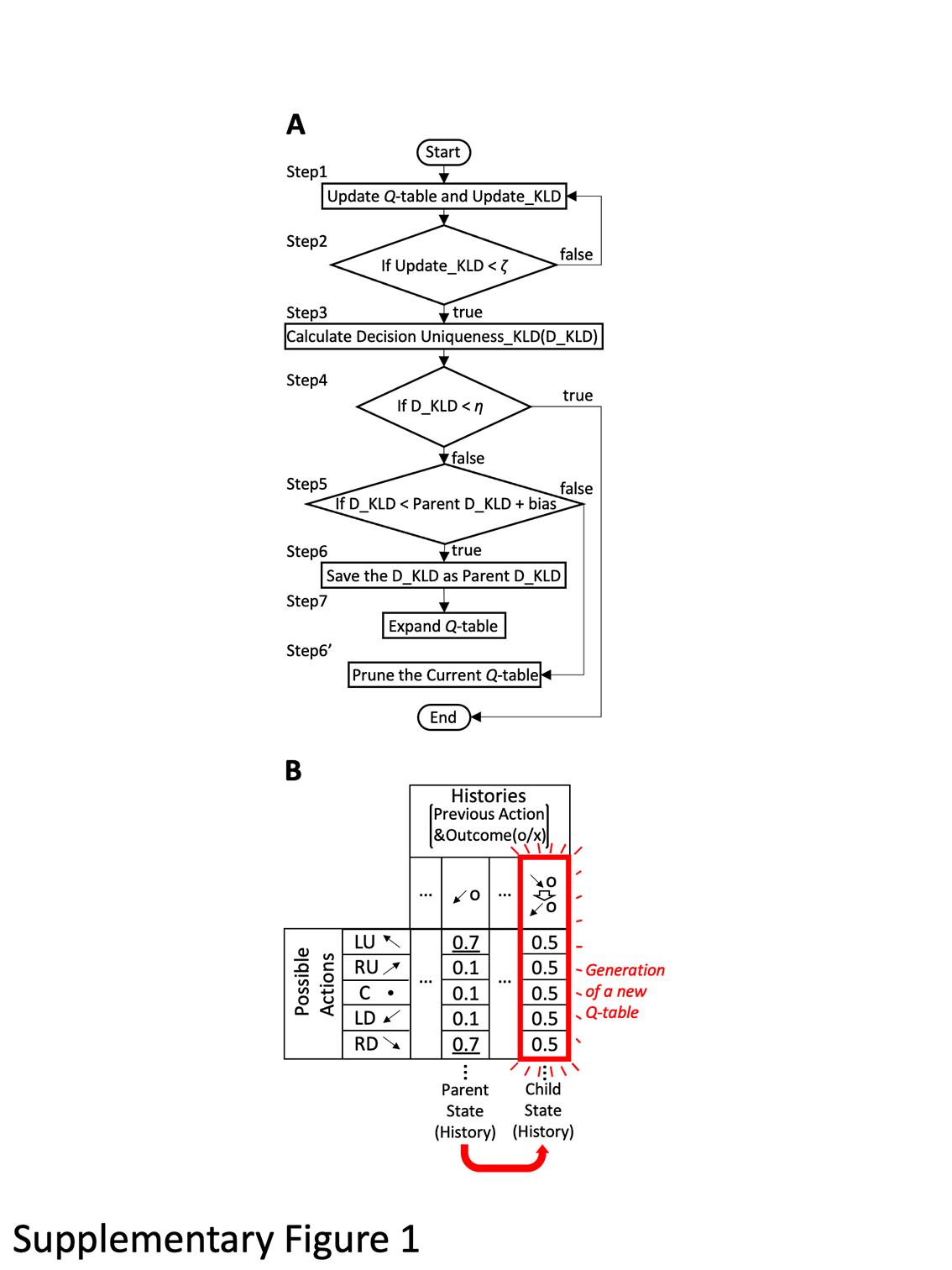

Supplement: Supplementary Figure 1 — Expansion and contraction of the history. (A) Flowchart of the expansion and contraction process. (B) An example of expansion of a history derived from the parent history in Q-table. The direction of the arrow represents the target that the agent looked at, and o and x represent the correct answer and error, respectively. The example in the figure shows that a new history is generated from the history that the agent looked at LD and was rewarded one trial ago, to the history that it looked at LD and was rewarded one trial ago after it looked at RD and was rewarded two trials ago. The numbers in the Q-table represent Q-values. The initial Q-value for each action is set to 0.5. [file Image_1.TIFF]

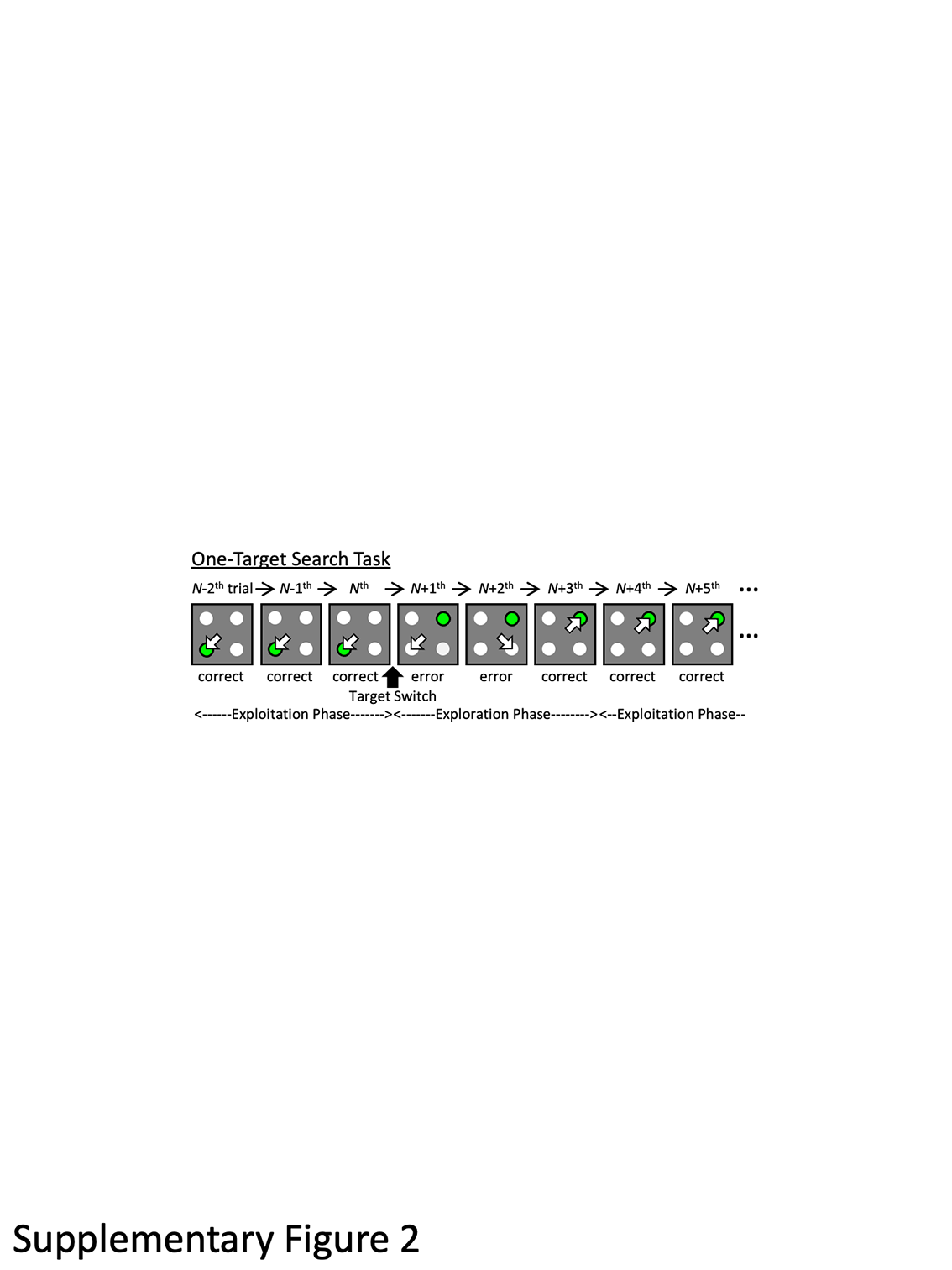

Supplement: Supplementary Figure 2 — A schematic example of a target switch in the one-target search task. The format is the same as the task shown in Figure 1B. [file Image_2.TIFF]
